# Screening anxiety via contrastive autobiographical recall

**DOI:** 10.3389/fdgth.2026.1798100

**Published:** 2026-05-25

**Authors:** Shamim Ibne Shahid, Mohammad Hassan Tayarani Najaran, Frank Förster, Volker Steuber

**Affiliations:** 1 Biocomputation Research Group, School of Physics, Engineering and Computer Science, University of Hertfordshire, Hatfield, United Kingdom; 2 Robotics Research Group, School of Physics, Engineering and Computer Science, University of Hertfordshire, Hatfield, United Kingdom

**Keywords:** anxiety screening, autobiographical memory recall, BERT, large language model, latent-space augmentation, PCA, support vector machines

## Abstract

**Introduction:**

Language offers a low-burden and scalable pathway for digital anxiety screening, particularly in telehealth or repeated-monitoring settings where spontaneous speech may already be available. This study introduces a contrastive autobiographical recall framework that uses short positive and negative personal memories to capture within person affective shifts in language. By modelling how the same individual expresses emotionally distinct experiences, the proposed approach aims to identify anxiety-related linguistic patterns that may not be captured from a single static text representation.

**Methods:**

A total of 156 participants completed a 5–7 minute spontaneous speech task involving positive and negative autobiographical memories. Anxiety status was defined using HAM-A scores, yielding non-anxious (*n* = 101) and anxious (*n* = 55) groups. Transcripts were segmented using Qwen-2.5-7B-Instruct as a deterministic constrained extractor, preserving only verbatim positive and negative spans alongside the complete transcript. Positive, negative, and complete narratives were encoded with frozen BERT model and combined with a contrast vector capturing within-person affective shift. Performance was evaluated using a leakage-safe leave-one-out cross-validation pipeline.

**Results:**

The proposed pipeline achieved 70% accuracy and 0.67 macro-F1 across leaveone-out folds, with stronger performance for non-anxious participants than anxious participants. Bootstrap confidence intervals were 0.62–0.77 for accuracy and 0.59–0.75 for macro-F1. Ablation analysis showed that the full composite representation provided the best balanced performance and strongest anxious-class detection. The method also outperformed BERT-based and lexicon-based baseline models.

**Discussion:**

These findings suggest that short autobiographical speech can provide a useful complementary signal for digital anxiety screening when modelled with contextual embeddings and within-person affective contrast. Latent-space augmentation supported learning in this small cohort without altering participant-authored language. However, anxious-class sensitivity was moderate, and HAM-A labels should be interpreted as screening rather than diagnostic labels. Further validation in larger and more diverse clinical cohorts is needed.

## 1 Introduction

Anxiety disorders are among the most common mental disorders worldwide and contribute substantially to disability and reduced quality of life (1–3). Because effective treatments exist and are recommended to be made available promptly, timely identification and referral are central to good care pathways (4). Yet, large-scale screening in routine settings remains difficult: structured diagnostic interviews are clinician-administered and time-intensive (even brief instruments typically require 15–20 min), and sustained monitoring is hard to maintain in community and primary-care contexts (3, 5–7). Brief validated screening questionnaires such as the GAD-7 are already widely used and can be delivered digitally. Our aim is not to replace such tools, but to investigate whether short spontaneous speech can provide a complementary, low-burden screening signal, particularly in settings such as repeated monitoring or telehealth workflows where such speech data are already available. These considerations motivate scalable *digital screening* approaches that can support triage and referral decisions when specialist resources are limited (8, 9).

Language is an attractive modality for digital screening because it is inexpensive to capture and closely coupled to cognitive and affective processes. A large body of psycholinguistic work shows that patterns of word use reflect psychological states (e.g., self-focus, affective tone, and cognitive processing) (10). In clinical and mental-health related text, anxiety has been associated with detectable linguistic signatures, including rigid or absolutist framing and other stylistic markers, although effects are heterogeneous across individuals and contexts (11, 12). In parallel, pretrained transformer encoders such as BERT provide high-capacity contextual representations that capture semantics beyond surface lexical counts (13). Recent work in Möell and Sand Aronsson (14) illustrates the potential of BERT-derived embeddings for mental health prediction, reporting high accuracy when models are trained and evaluated on large language model–generated self-report narratives in a synthetic-only settings. However, the authors emphasise that such performance is measured within a synthetic distribution and does not establish generalisation to real participant-produced language, reinforcing the need for careful evaluation in modest, real-world cohorts. This choice is also consistent with prior mental-health NLP studies that leverage BERT-based representations for prediction from language, for example using BERT-driven sentiment modelling in psychiatric interviews to relate language markers to depressive symptom severity (15). However, applying such rich representations in modest clinical cohorts raises a key methodological risk. In human behavioral datasets with limited sample sizes, predictive performance estimates can be highly variable and may become overly optimistic when evaluation permits leakage between training and test data (16), or uses inappropriate cross-validation schemes such as sample-level rather than participant-level splitting (17).

A second challenge is that common text augmentation methods (e.g., synonym replacement, paraphrasing, back-translation) may be poorly aligned with clinically meaningful autobiographical narratives. These techniques are widely used in general NLP (18, 19), but they can introduce semantic drift or stylistic artifacts that weaken label integrity and interpretability—a concern that is especially salient when the precise wording, emphasis, and spontaneous framing are part of the signal of interest (18, 20). For mental health applications, where auditability and transparent linkage between model inputs and participant-authored text are often required, it can be preferable to improve robustness *without* generating new text (9, 21).

In this study, we investigate language-based screening of anxiety using a *contrastive autobiographical memory recall* paradigm in which participants produce both positive and negative spontaneous memories within a single session. Emotionally valenced recall is known to modulate linguistic and affective expression in autobiographical narration, providing an opportunity to model *within-person* affective shifts in addition to between-person differences (22, 23). By explicitly comparing how the same individual recounts positive vs. negative material, we aim to reduce sensitivity to stable confounds (e.g., topic choice, verbosity, idiolect) while amplifying anxiety-linked appraisal or emotional reactivity expressed through language (10, 12).

To exploit this property while maintaining traceability, we decompose each transcript into valence-specific excerpts and retain the full narrative as a reference view. Our segmentation is conservative and auditable: it selects only verbatim spans from the participant transcript and does not generate new language. This design aligns with broader expectations for transparency and traceability in clinical AI systems, where users and regulators require clear links between model behaviour and the underlying evidence (21, 24–26). Each text view is encoded using a pretrained BERT model (bert-large-uncased) (13) with attention-masked mean pooling. We then construct a composite representation by concatenating positive, negative, and complete narrative embeddings together with a difference vector that captures within-person affective shift.

To mitigate overfitting under a small sample size, we apply augmentation in a reduced latent space rather than perturbing raw text. This choice was motivated by two considerations. First, prior work has shown that simple transformations in learned feature space can serve as a generic augmentation strategy for generating plausible synthetic examples and improving downstream performance (27). Second, latent-space augmentation is particularly useful when transformations are difficult to define directly in the input space, as is often the case when semantic structure must be preserved (28). We use leave-one-out cross-validation (LOOCV) in a participant-wise manner, holding out all data from one participant per fold. All preprocessing is fit using training-only statistics to preserve strict train–test separation. Classification is performed using an RBF-kernel support vector machine (SVM), a margin-based classifier that uses kernels (including the Gaussian/RBF kernel) to model non-linear decision boundaries (29, 30). SVMs are widely used in biomedical prediction settings and are often favoured in high-dimensional, limited-sample regimes (31, 32).

Overall, we present an empirically evaluated, language-based approach to anxiety screening in a small cohort. Specifically, we:

1. Construct participant-level representations from positive, negative, and complete autobiographical narratives using pretrained contextual language embeddings, including a contrastive shift term to capture within-person differences across recall contexts (22).
2. Evaluate the model performance in a leakage-safe leave-one-out cross-validation (LOOCV) pipeline (i.e., standardisation and PCA fitting are estimated on the training fold and then applied to the held-out sample). Class-conditional Gaussian augmentation is performed in the PCA-projected latent space, increasing effective training density to mitigate overfitting under limited sample sizes.
3. Complement predictive evaluation with exploratory analyses that examine how valence-conditioned narrative views (positive, negative, complete, and contrast) affect model performance and anxious-vs.-non-anxious separability.
4. Analyse anxious vs. non-anxious group differences in interpretable lexicon-based linguistic markers (10, 11) and motivate the need for contextualised language embeddings beyond heuristic counts.

## 2 Materials and methods

### 2.1 Data collection

#### 2.1.1 Experimental procedure and acquisition

The study was approved by the University of Hertfordshire under protocol number SPECS/SF/UH/05493; see Appendix B. Participants completed a single spontaneous speech recording lasting approximately 5–7 min. During the recording, they were asked to describe a personally meaningful positive memory and a personally meaningful negative memory. This autobiographical recall approach was selected because emotional recollection can systematically influence *what* people say and *how* they say it, yielding measurable shifts in linguistic content and style that reflect underlying psychological processes (10, 23, 33). In particular, clinically relevant language markers may include changes in affective wording, self-focus and cognitive/appraisal language (10, 33), as well as more rigid or absolutist framing that has been linked to anxiety-related distress (11).

Audio was recorded using a standard consumer-grade device in a quiet environment. In the present study, recordings were transcribed using the AssemblyAI Speech-to-Text API (34), and only the text modality was retained for analysis. No constraints were imposed on linguistic content, allowing speech to remain natural and participant-driven. All recordings were stored securely and used exclusively for research purposes.

#### 2.1.2 Anxiety assessment

After completing the autobiographical recall recording, participants were assessed using the Hamilton Anxiety Rating Scale (HAM-A), a clinically established measure of anxiety severity that covers both psychological and somatic symptom domains (35). Total HAM-A scores were then converted into a binary screening label using commonly adopted severity thresholds in the clinical literature (36, 37). Specifically, participants with scores <18 (no/mild anxiety) were assigned to the *non-anxious* group (n=101), whereas those with scores ≥18 (moderate/severe anxiety) were assigned to the *anxious* group (n=55). These labels served as the reference labels for all downstream machine learning analyses.

### 2.2 Valence-specific text segmentation

To support clinically meaningful digital screening from spontaneous speech, we decomposed each participant’s transcript into *valence-specific* excerpts reflecting positive and negative affect, while also retaining the *complete narrative* as a neutral reference. This segmentation step was designed to be **conservative and auditable**: the pipeline *selects* participant-authored phrases rather than generating new text, thereby preserving the original wording and reducing the risk of clinically misleading alterations.

#### 2.2.1 LLM-constrained span extraction

**Figure 1:**
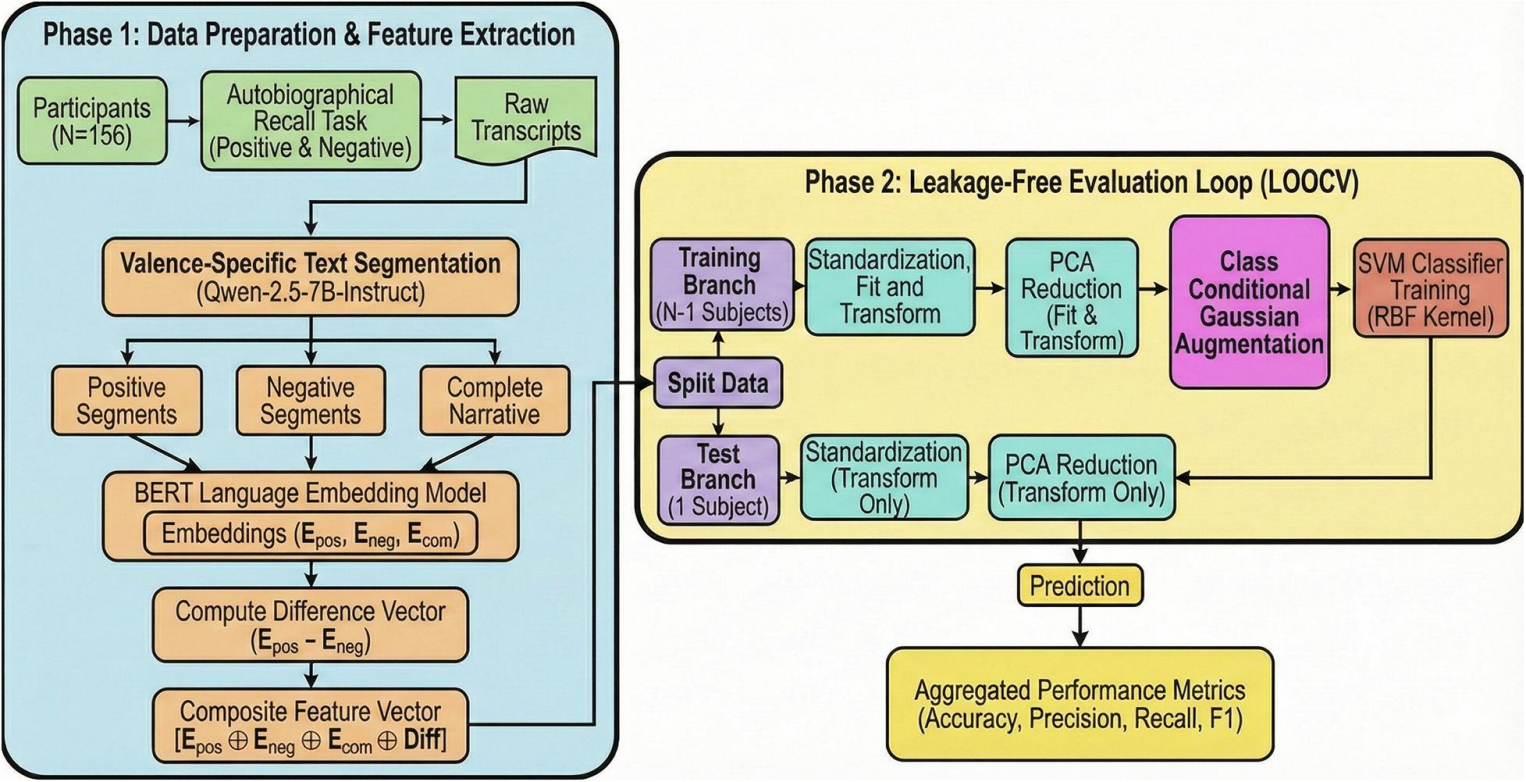
Overview of the proposed anxiety screening pipeline. Participants complete an autobiographical recall task producing positive, negative, and neutral narratives, which are transcribed and processed via valence-specific text segmentation. Each segment is encoded using a BERT-based language embedding model and combined into a composite contrastive feature representation. Model training and evaluation are conducted within a leakage-safe Leave-One-Out Cross-Validation (LOOCV) framework, incorporating PCA-based dimensionality reduction, latent Gaussian data augmentation, and an SVM classifier. Performance metrics are aggregated across all folds.

We used the Qwen-2.5-7B-Instruct (38) large language model as a constrained extractor to identify sentiment-bearing sentences within the raw transcript T, as shown in Figure 1. Decoding was performed deterministically (temperature = 0.0). The model was instructed to output strict JSON only containing two fields, “positive” and “negative”, each a list of sentences. Extraction was governed by a hard **verbatim constraint**, requiring each extracted sentence to be an exact substring (direct quotation) of the source transcript. This formulation encourages the model to function as a structured filter over the participant’s narrative rather than a summariser, which aligns with interpretability expectations in digital health applications.

#### 2.2.2 Validation and traceability

To prevent hallucinated or paraphrased content from entering the modelling stage, we applied a strict multi-stage quality control procedure to all extracted spans:

1. **Schema enforcement:** The generated output was parsed to recover a valid JSON object, and any extraneous tokens outside the predefined schema were discarded.
2. **Verbatim verification:** Each extracted span s was retained only if it appeared verbatim in the original transcript (s∈T). Spans failing this exact substring check were excluded from further analysis.

This validation pipeline ensures full traceability between extracted spans and the source text, and guarantees that all downstream representations are grounded exclusively in participant-produced language.

#### 2.2.3 Valence documents, BERT featurisation, and composite feature construction

Validated positive sentences were concatenated (in original chronological order) to form a *Positive* document $D_{\mathrm{pos}}$, validated negative spans were concatenated to form a *Negative* document $D_{\mathrm{neg}}$, and the unaltered transcript was retained as the *Complete* narrative $D_{\mathrm{com}}$. Each document $D\in \{D_{\mathrm{pos}},D_{\mathrm{neg}},D_{\mathrm{com}}\}$ was then encoded using a pretrained BERT encoder (bert-large-uncased) (13) operating in inference mode (no fine-tuning).

Let the final hidden states be $H\in R^{T\times d}$ for a tokenised sequence of length T and hidden size d=1024, corresponding to the output dimensionality of bert-large-uncased, with attention mask $m\in \{0,1{\}}^{T}$ indicating valid (non-padding) tokens. Following the general pooling strategy used in prior work, where token-level BERT representations are aggregated by mean pooling to obtain a fixed-dimensional text embedding (14), we derived each document representation using attention-masked mean pooling, as defined in Equation 1, so that only valid tokens contributed to the pooled vector:

$$E(D)=\frac{\sum_{t=1}^{T}m_{t}H_{t}}{\sum_{t=1}^{T}m_{t}}\in R^{d}.$$ (1)

This yields a single fixed-dimensional embedding for each document while excluding padding tokens from the aggregation. In implementation, tokenisation used padding and truncation with a maximum sequence length of 512 tokens. This produced participant-level embeddings, as shown in Equation 2:

$$E_{\mathrm{pos}}=E(D_{\mathrm{pos}}),\;E_{\mathrm{neg}}=E(D_{\mathrm{neg}}),\;E_{\mathrm{com}}=E(D_{\mathrm{com}}),\;E_{\mathrm{pos}},E_{\mathrm{neg}},E_{\mathrm{com}}\in R^{1024}.$$ (2)

To explicitly model affective contrast within an individual, we further computed a *difference vector*, as defined in Equation 3:

$$E_{\Delta }=E_{\mathrm{pos}}-E_{\mathrm{neg}}\in R^{d}=R^{1024}.$$ (3)

Finally, we formed a composite participant representation by concatenating the three valence-conditioned embeddings and the difference vector, as defined in Equation 4:

$$X=[\;E_{\mathrm{pos}}⊕E_{\mathrm{neg}}⊕E_{\mathrm{com}}⊕E_{\Delta }\;]\in R^{4d}=R^{4096},$$ (4)

where ⊕ denotes vector concatenation. This composite feature vector X was used as input to the downstream LOOCV evaluation pipeline (standardisation, PCA, latent augmentation, and SVM classification).

### 2.3 PCA projection and latent Gaussian augmentation

To mitigate overfitting under small sample size and to stabilise the classifier decision boundary, we apply augmentation in a reduced latent space rather than perturbing raw text, as outlined in Algorithm 1. For each LOOCV iteration, the training fold $\{(X_{i},y_{i}){\}}_{i=1}^{N-1}$ is first standardised and then projected into a fixed K-dimensional PCA space (K=112 in all our experiments):

Algorithm 1 **LOOCV with leakage-free preprocessing and data augmentation.**

**Require:** Dataset $X\in R^{N\times D}$, Labels $y\in \{0,1{\}}^{N}$

**Ensure:** Predictions $\hat{Y}=\{{\hat{y}}_{1},\ldots ,{\hat{y}}_{N}\}$

1: **for** sample i=1 toN **do**

2:   **1. Data Splitting**

3:   $x_{test}\leftarrow X[i]$           ▹ Hold-out sample

4:   $y_{test}\leftarrow y[i]$

5:   $X_{train}\leftarrow X\setminus \{X[i]\}$           ▹ Remaining N−1 samples

6:   $y_{train}\leftarrow y\setminus \{y[i]\}$

7:   **2. Standardization (Z-Score)**

8:   Compute $\mu_{train},\sigma_{train}$ from $X_{train}$           ▹ Fit scaler on Train only

9:   $X_{train}^{\prime }\leftarrow \frac{X_{train}-\mu_{train}}{\sigma_{train}}$           ▹ Transform Train

10:   $x_{test}^{\prime }\leftarrow \frac{x_{test}-\mu_{train}}{\sigma_{train}}$           ▹ Transform Test using Train stats

11:   **3. Dimensionality Reduction (PCA)**

12:   Learn projection matrix $W\in R^{D\times K}$ from $X_{train}^{\prime }$     ▹ Fit PCA on Train only

13:   $Z_{train}\leftarrow X_{train}^{\prime }\cdot W$           ▹ Project Train to latent space

14:   $z_{test}\leftarrow x_{test}^{\prime }\cdot W$           ▹ Project Test to latent space

15:   **4. Gausssian Data Augmentation (Train fold only)**

16:   $Z_{\text{syn}}\leftarrow \emptyset ,y_{\text{syn}}\leftarrow \emptyset$

17:   **for** classc∈{0,1} **do**

18:    $Z_{c}\leftarrow \{z\in Z_{\text{train}}\;:\;y_{\text{train}}=c\}$           ▹ Class-conditional latent set

19:   $\sigma_{c}\leftarrow \mathrm{std}(Z_{c})$           ▹ Per-dimension std in $R^{K}$

20:   Choose number of synthetic samples $M_{c}$           ▹ e.g., to balance classes

21:   **for** m=1 to$M_{c}$ **do**

22:    Sample base point $z_{base}∼\mathrm{Uniform}(Z_{c})$

23:    Sample noise $\epsilon ∼N(0,\lambda^{2}\mathrm{diag}(\sigma_{c}^{2}))$           $▹\;\epsilon \in R^{K}$

24:    $z_{\text{new}}\leftarrow z_{\text{base}}+\epsilon$

25:    $Z_{\text{syn}}\leftarrow Z_{\text{syn}}\cup \{z_{\text{new}}\}$

26:    $y_{\text{syn}}\leftarrow y_{\text{syn}}\cup \{c\}$

27:   **end** **for**

28:  **end** **for**

29:  $Z_{\text{aug}}\leftarrow Z_{\text{train}}\cup Z_{\text{syn}}$

30:  $y_{\text{aug}}\leftarrow y_{\text{train}}\cup y_{\text{syn}}$

31:  **5. Classification**

32:  Train SVM on $\left(Z_{\text{aug}},y_{\text{aug}}\right)$

33:  ${\hat{y}}_{i}\leftarrow \mathrm{SV}\;M(z_{\text{test}})$           ▹ Predict label for held-out sample

34:  **end** **for**

$$Z_{i}=\mathrm{PCA}\;\left(\mathrm{zscore}\;(X_{i})\right),\;Z_{i}\in R^{K}.$$

Let $Z_{\text{train}}$ and $y_{\text{train}}$ denote the latent training set and labels in the current fold. For each class c∈{0,1}, we compute the per-dimension standard deviation vector in latent space:

$$\sigma_{c}=\mathrm{std}\;\;\left(\{Z_{i}\;\;:\;\;y_{i}=c\}\right)\in R^{K},$$

with zero-variance dimensions floored to a small constant for numerical stability. Synthetic samples are then generated by adding class-conditional Gaussian noise to real latent points. For a base point $z\in R^{K}$ from class c, we sample

$$\epsilon ∼N\;\;\left(0,\lambda^{2}\;\mathrm{diag}\;(\sigma_{c}^{2})\right),\;z_{\text{syn}}=z+\epsilon ,$$

where λ controls augmentation strength (set to λ=0.2). We generate $n_{\text{aug}}=10$ synthetic variants per real training sample, yielding an augmented training set $\left(Z_{\text{aug}},y_{\text{aug}}\right)$ used for classifier training. Augmentation is applied only to the training fold to maintain strict separation between train and test data within LOOCV.

### 2.4 Support vector machine (SVM)

Classification was performed using a Support Vector Machine (SVM) with a radial basis function (RBF) kernel (29, 30) on the PCA-reduced feature set. Given the modest cohort size and the use of leave-one-out cross-validation (LOOCV), extensive hyperparameter tuning was avoided, as it could introduce additional variance and increase the risk of optimistic generalisation estimates. We therefore adopted stable, commonly used settings, using gamma=scale to adapt the kernel width automatically to the variance of the standardised feature space and setting the regularisation parameter to C=5.0 as a moderate compromise between maximising the decision margin and preserving sensitivity to individual samples. We also verified that the results were robust across nearby C values; these additional analyses are reported in Appendix C. To address class imbalance between anxious and non-anxious participants, class-weighted training was applied so that both groups contributed proportionally to the optimisation objective.

### 2.5 Evaluation metrics

Model performance was evaluated using accuracy, precision, recall, and F1-score. In addition to overall accuracy, both macro-averaged and weighted-averaged metrics were reported to provide a balanced assessment across classes. These metrics collectively capture sensitivity to anxiety-related patterns while accounting for class imbalance, yielding a comprehensive evaluation of model performance. Although ROC-AUC is commonly reported in clinical machine-learning studies, we focused here on threshold-specific metrics because the analysis was conducted under a fixed decision rule within the LOOCV pipeline. Accordingly, we report accuracy, precision, recall, and F1-score as the primary evaluation measures.

## 3 Results

Model performance under the LOOCV protocol is summarised in Table 1. Overall, the proposed pipeline achieved an accuracy of 0.70 and a macro-averaged F1-score of 0.67, suggesting that the approach provides meaningful discrimination in this limited-sample digital screening setting.

**Table 1:** Classification performance aggregated across LOOCV folds.

Class	Precision	Recall	F1-score	Support
Non-Anxious	0.77	0.76	0.77	101
Anxious	0.57	0.58	0.58	55
Accuracy			0.70	156
Macro Avg	0.67	0.67	0.67	156
Weighted Avg	0.69	0.70	0.69	156

Class-wise performance indicates higher reliability for identifying *Non-Anxious* participants (precision = 0.77, recall = 0.76; F1 = 0.77) than for detecting the *Anxious* group (precision = 0.57, recall = 0.58; F1 = 0.58). This pattern is consistent with the class distribution (101 vs. 55) and with heterogeneity in how anxiety is expressed in short autobiographical narratives, particularly for participants near the clinical threshold.

To characterise uncertainty in the headline results, we computed bootstrap 95% confidence intervals (CIs) from the aggregated out-of-fold LOOCV predictions (Table 2). Accuracy was 0.70 (95% CI: 0.62–0.77) and macro-F1 was 0.67 (95% CI: 0.59–0.75), indicating that performance remains above chance across resampled cohorts. For completeness, Table 2 also reports CIs for positive-class (anxious) precision, recall, and F1 (F1 = 0.58, 95% CI: 0.46–0.68; precision = 0.57, 95% CI: 0.44–0.70; recall = 0.58, 95% CI: 0.45–0.71), which reflect the expected variability in anxious-case detection under resampling.

**Table 2:** Bootstrap 95% confidence intervals (CIs) for key metrics computed from LOOCV out-of-fold predictions.

Metric	Estimate	95% CI
Accuracy	0.70	[0.62, 0.77]
Macro-F1	0.67	[0.59, 0.75]
F1 (Anxious)	0.58	[0.46, 0.68]
Precision (Anxious)	0.57	[0.44, 0.70]
Recall (Anxious)	0.58	[0.45, 0.71]

Taken together, these results support the feasibility of the proposed contrastive recall-based pipeline as an initial screening approach, while underscoring the need for further validation in larger and more diverse cohorts.

## 4 Ablation study

To examine the contribution of each representation in the proposed contrastive autobiographical recall framework, we conducted a controlled ablation study in which **only the input representation X was varied**, while **all other stages of the pipeline were kept identical**: leakage-safe LOOCV, fold-wise standardisation and PCA (fit on the training fold only), latent-space class-conditional Gaussian augmentation applied to the training fold only, and the same downstream SVM classifier and evaluation procedure. The candidate representations were derived from the four components defined in our feature construction: the positive narrative embedding $E_{\text{\textbackslash{},pos}}$, the negative narrative embedding $E_{\text{neg}}$, the complete narrative embedding $E_{\text{com}}$, and the contrastive shift vector $E_{\Delta }=E_{\text{\textbackslash{},pos}}-E_{\text{neg}}$.

### 4.1 Ablation settings

We evaluated three groups of feature configurations. First, we tested each representation individually: **Negative only** ($X=E_{\text{neg}}$), **Positive only** ($X=E_{\text{\textbackslash{},pos}}$), **Complete only** ($X=E_{\text{com}}$), and **Contrast only** ($X=E_{\Delta }$). Second, we evaluated all six pairwise combinations of these components: $E_{\text{\textbackslash{},pos}}⊕E_{\text{neg}}$, $E_{\text{\textbackslash{},pos}}⊕E_{\text{com}}$, $E_{\text{\textbackslash{},pos}}⊕E_{\Delta }$, $E_{\text{neg}}⊕E_{\text{com}}$, $E_{\text{neg}}⊕E_{\Delta }$, and $E_{\text{com}}⊕E_{\Delta }$. Finally, we evaluated the **full composite** representation, $X=E_{\text{\textbackslash{},pos}}⊕E_{\text{neg}}⊕E_{\text{com}}⊕E_{\Delta }$.

### 4.2 Results

Table 3 reports LOOCV-aggregated performance across all single-view, pairwise, and full-composite settings. Among the single-view representations, the **complete narrative** ($E_{\text{com}}$) achieved the strongest performance (Acc.=0.65, Macro-F1=0.62, Recall${}_{1}$=0.51, F1${}_{1}$=0.51), whereas the **negative-only** representation performed worst (Acc. = 0.52, Macro-F1 = 0.49). Among the pairwise combinations, $E_{\text{com}}⊕E_{\Delta }$ gave the highest accuracy (0.71) and the strongest macro-F1 (0.65), indicating that the complete narrative and the contrastive shift provide complementary information when combined. However, the **full composite representation** still yielded the best overall balanced performance, achieving the highest macro-F1 (**0.67**) as well as the strongest anxious-class detection (**Recall${}_{1}$ = 0.58**, **F1${}_{1}$ = 0.58**).

**Table 3:** Ablation over single-view, pairwise, and full composite representations under the LOOCV pipeline.

Input representation	Acc.	Macro-F1	Recall${}_{1}$	F1${}_{1}$
Single-view representations				
Negative ($E_{\text{neg}}$)	0.52	0.49	0.38	0.36
Positive ($E_{\text{\textbackslash{},pos}}$)	0.61	0.58	0.49	0.47
Complete ($E_{\text{com}}$)	0.65	0.62	0.51	0.51
Contrast ($E_{\Delta }$)	0.65	0.61	0.47	0.49
Pairwise combinations				
$E_{\text{\textbackslash{},pos}}⊕E_{\text{neg}}$	0.65	0.59	0.38	0.43
$E_{\text{\textbackslash{},pos}}⊕E_{\text{com}}$	0.65	0.57	0.33	0.40
$E_{\text{\textbackslash{},pos}}⊕E_{\Delta }$	0.65	0.58	0.35	0.41
$E_{\text{neg}}⊕E_{\text{com}}$	0.66	0.59	0.36	0.43
$E_{\text{neg}}⊕E_{\Delta }$	0.65	0.58	0.35	0.41
$E_{\text{com}}⊕E_{\Delta }$	**0.71**	0.65	0.40	0.49
Full composite				
** $E_{\text{\textbackslash{},pos}}⊕E_{\text{neg}}⊕E_{\text{com}}⊕E_{\Delta }$ **	0.70	**0.67**	**0.58**	**0.58**

Metrics are shown to two decimals.

Metrics are shown to two decimals; bold values indicate the best-performing result for each metric.

### 4.3 Interpretation

Three main patterns emerge from this ablation. First, the **complete narrative** ($E_{\text{com}}$) is the strongest individual representation, suggesting that anxiety-relevant cues are distributed across the broader autobiographical account rather than being confined to explicitly positive or negative excerpts alone. Second, the strong performance of the pairwise combination $E_{\text{com}}⊕E_{\Delta }$ indicates that the contrastive shift signal is most informative when anchored by the full narrative context, rather than used in isolation. Third, although some pairwise combinations improved over single-view representations, the **full composite model** remained the most effective overall, especially for anxious-class detection. This supports the central design choice of the proposed framework: modelling both the absolute content of the autobiographical narratives and the within-person affective contrast between positive and negative recall provides the most balanced screening performance.

### 4.4 Comparison with BERT-based baselines

To contextualise the benefit of the proposed contrastive construction, we compared our full pipeline against two BERT-based baselines under the same LOOCV evaluation protocol. First, we evaluated an *embedding-only* baseline that uses frozen bert-large-uncased embeddings of the complete narrative and trains a logistic regression classifier on the resulting representation. Second, we evaluated an *end-to-end fine-tuned BERT* baseline in which bert-large-uncased is fine-tuned with a linear classification head.

Table 4 shows that the proposed method achieves the strongest overall performance (**Accuracy = 0.70**, **Macro-F1 = 0.67**), improving over both the embedding-only logistic regression baseline (Accuracy = 0.62, Macro-F1 = 0.61) and the fine-tuned BERT baseline (Accuracy = 0.64, Macro-F1 = 0.49). Relative to embedding-only logistic regression, our approach yields a clear gain in balanced performance (Macro-F1: 0.67 vs. 0.61), indicating that explicitly modelling valence-conditioned views and within-person contrast provides additional discriminative signal beyond using BERT embeddings alone.

**Table 4:** Performance comparison under participant-wise LOOCV (leave-one-participant-out).

Method	Accuracy	Macro-F1	Precision	Recall
BERT fine-tuned (end-to-end)	0.64	0.49	0.57	0.53
BERT features + Logistic Regression	0.62	0.61	0.62	0.63
**Proposed Method**	0.70	0.67	0.67	0.67

Notably, fine-tuning BERT end-to-end did not improve results in this small-sample setting: although overall accuracy was 0.64, Macro-F1 dropped to 0.49, reflecting poorer class-balanced performance. This pattern is consistent with the higher variance and overfitting risk of end-to-end fine-tuning under participant-wise LOOCV with modest cohort size, where the model can over-specialise to majority-class patterns and yield unstable minority-class performance. In contrast, the proposed pipeline maintains both higher precision and recall in macro-average terms (both 0.67), suggesting more consistent discrimination across anxious and non-anxious participants.

### 4.5 Why contextual language embeddings are necessary beyond heuristic markers

For digital screening, it is important to test whether anxiety status can be inferred using simple and clinically transparent lexical heuristics—such as self-focus, uncertainty cues, affect/anxiety lexicons, or absolutist framing (10, 11)—rather than relying on high-dimensional contextual representations . To examine this directly, we computed the 12 lexicon-based biomarkers defined in Appendix A on each participant’s full transcript and used these markers as a standalone feature set. We then evaluated three standard classifiers under the same leakage-safe leave-one-out (LOOCV) protocol used throughout the paper (i.e., all preprocessing is fit on the training fold only). Table 5 reports aggregated LOOCV performance.

**Table 5:** LOOCV performance of baseline classifiers trained on the 12 lexicon-based linguistic biomarkers (Appendix A).

Model	Accuracy	Macro F1
RBF-SVM	0.60	0.58
MLP	0.62	0.59
Random Forest	0.61	0.50

Overall, lexicon-biomarker baselines provide only moderate discrimination (accuracy ≈0.60−0.62; macro F1 ≈0.50−0.59). The MLP yields the strongest overall balance (Macro-F1 = 0.59), with the RBF-SVM close behind (Macro-F1 = 0.58). In contrast, the Random Forest shows poorer class balance (Macro-F1 = 0.50), suggesting that tree-based decision boundaries can be unstable when trained on a small, low-dimensional marker set under class imbalance.

**Figure 2:**
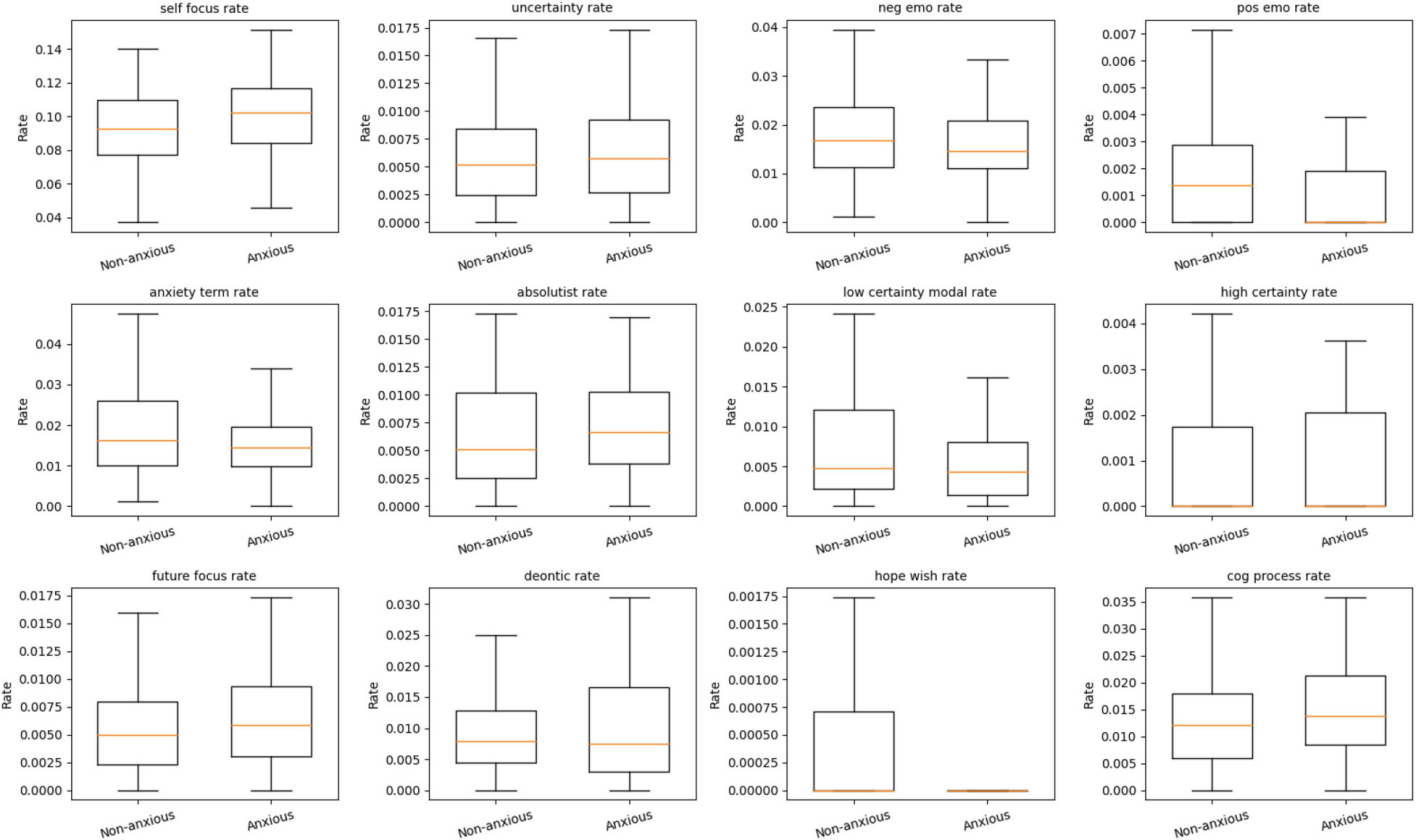
Distribution of lexicon-based linguistic biomarkers in anxious vs. non-anxious participants. Boxplots show rate-normalised marker frequencies computed on each participant’s *full transcript* (Appendix A). Several markers exhibit small shifts in median between groups (e.g., self-focus, uncertainty, positive/negative affect, and absolutist wording), but substantial overlap remains across participants, highlighting heterogeneity in how anxiety is expressed through language.

Figure 2 helps interpret these results. Several biomarkers show modest group-level shifts in central tendency, including slightly higher self-focus and uncertainty rates in the anxious group and lower positive emotion rates, alongside small changes in negative affect and absolutist wording. However, the dominant pattern across all markers is substantial between-participant variability and strong overlap between anxious and non-anxious groups. In a screening setting, this overlap limits individual-level separability: even when medians differ, many participants from both groups occupy similar value ranges, constraining the performance of any classifier that relies only on surface-count summaries.

This motivates the use of contextual language embeddings (13) in our main pipeline. Lexicon rates capture *what* categories of words appear, but they are largely insensitive to *how* language is used in context: negation and intensification, attribution (e.g., “I think” vs. “it is”), pragmatic intent, and narrative/discourse structure. These contextual factors can be critical in autobiographical recall, where clinically relevant signals may be expressed implicitly rather than through direct symptom terms. By encoding meaning in context, LLM based embeddings can represent compositional and discourse-level information that is not recoverable from lexicon counts alone. Accordingly, we treat lexicon-based biomarkers as an interpretable baseline and use contextual embeddings as the primary representation under the same leakage-safe evaluation protocol.

## 5 Conclusion

This study introduced a novel framework for digital anxiety screening that leverages the linguistic contrast between positive and negative autobiographical recall. By utilizing a deterministic, auditable LLM-based extraction process to isolate valence-specific content without altering participant wording, combined with latent-space data augmentation to mitigate overfitting, the proposed pipeline achieved a classification accuracy of 70% and a macro-F1 of 0.67 in a leakage-safe evaluation.

Our findings support three key conclusions. First, the ablation study demonstrates that anxiety screening is most effective when modelling the *shift* in linguistic representation between emotional contexts. The composite feature representation—combining positive, negative, and complete narratives with a contrastive difference vector—outperformed any single narrative view, confirming that the interplay between how an individual frames positive vs. negative experiences contains a unique diagnostic signal. Second, the comparison with lexicon-based biomarkers reveals that while heuristic features (e.g., self-focus, absolutist words) show group-level trends, they lack the individual-level discriminative power provided by high-dimensional contextual embeddings. Finally, the application of class-conditional Gaussian augmentation in the PCA-projected latent space suggests that small clinical datasets can be effectively modelled without resorting to generative text augmentation, which risks introducing semantic drift or hallucinations.

While these results are promising, limitations remain regarding the asymmetric sensitivity between anxious and non-anxious classes. From a clinical perspective, the stronger performance for the Non-Anxious group suggests that, in its current form, the model may be better suited as a complementary decision-support tool alongside established screening instruments rather than as a standalone primary screening method. In particular, it may be more useful for supporting risk stratification or follow-up assessment than for first-line case finding, since the detection of anxious participants remained comparatively modest. The current model favours specificity, which is valuable for reducing false positives but requires calibration for screening contexts where sensitivity is paramount.

Additionally, anxiety labels in the present study were derived from HAM-A scores collected in a research setting without clinician administration, and no additional validated anxiety questionnaire or structured diagnostic interview was included for convergent validation. Accordingly, the reference labels should not be interpreted as equivalent to formal clinical diagnosis, and the present findings should be viewed as preliminary evidence for digital screening rather than diagnostic classification. Moreover, the autobiographical recall paradigm may capture broader emotional responding in addition to anxiety.

## Data Availability

Due to compliance with GDPR-UK protections of personal identifiable data, we are unable to make the audio recordings of participants used within this study available to the wider research community. Requests to access the datasets should be directed to the corresponding author/s.

## Appendix

### A Lexicon-based linguistic biomarker quantification

This appendix specifies the procedure used to compute the 12 lexicon-based linguistic biomarkers visualised in Figure 2 and used in the heuristic baseline experiments (Table 5). Biomarkers are computed on the **complete narrative text** $D_{\text{com}}$, i.e., the participant’s full transcript.

### A.1 Tokenisation and normalisation

For $D_{\text{com}}$, we lowercased the text and tokenised it using a lightweight word regex (letters and apostrophes), yielding a sequence of tokens $\left\{w_{1},\ldots ,w_{n}\right\}$ with length n. For any lexicon L, the corresponding rate-normalised feature is defined using Appendix A1

$$r_{L}(D_{\text{com}})=\frac{1}{n}\sum_{i=1}^{n}I[w_{i}\in L],$$ (A1)

with $r_{L}(D_{\text{com}})=0$ when n=0 (empty transcript). This rate normalisation controls for differences in verbosity across participants.

Some biomarkers additionally include a small set of common multi-word cues (phrases). For such biomarkers, we compute a combined rate using Appendix A2

$$r_{L,P}(D_{\text{com}})=\frac{c_{L}(D_{\text{com}})+c_{P}(D_{\text{com}})}{n},$$ (A2)

where $c_{L}(D_{\text{com}})$ is the token-level lexicon count and $c_{P}(D_{\text{com}})$ is the total number of matched phrases in the lowercased document (counted using exact word-boundary matches).

### A.2 Biomarker definitions (12 features)

Using the tokenisation and normalisation above, we computed the following 12 biomarkers of anxiety on $D_{\text{com}}$:

1. **Self-focus rate** ($r_{\text{self}}$): first-person singular pronouns (e.g., *i, me, my, mine, myself*) (10, 33).
2. **Uncertainty/hedging rate** ($r_{\text{unc}}$): uncertainty words plus hedge phrases (e.g., *maybe, perhaps, seems* and phrases such as *i think, i don’t know, not sure*); computed as $r_{L,P}(D_{\text{com}})$ (10, 33).
3. **Negative emotion rate** ($r_{\text{negemo}}$): negative affect lexicon (e.g., *sad, upset, angry, scared, nervous*) (10, 33).
4. **Positive emotion rate** ($r_{\text{\textbackslash{},posemo}}$): positive affect lexicon (e.g., *happy, calm, peaceful, grateful, confident*) (10, 33).
5. **Anxiety-term rate** ($r_{\text{anx}}$): anxiety-related terms (e.g., *anxious, worry, fear, panic, uneasy, overwhelmed*) (12, 39).
6. **Absolutist rate** ($r_{\text{abs}}$): rigid/all-or-nothing words (e.g., *always, never, nothing, everything, completely, totally*) (11).
7. **Low-certainty modal rate** ($r_{\text{lowmod}}$): tentative modals (e.g., *might, could, may, perhaps, possibly*) (10).
8. **High-certainty rate** ($r_{\text{highcert}}$): certainty markers (e.g., *definitely, certainly, surely*) (10).
9. **Future-focus rate** ($r_{\text{\textbackslash{},future}}$): future-oriented markers (e.g., *will, tomorrow, next, later, future*) (10).
10. **Deontic/obligation rate** ($r_{\text{deon}}$): obligation/pressure tokens plus common phrases (e.g., *must, should, need* and phrases such as *have to, need to*); computed as $r_{L,P}(D_{\text{com}})$ (10).
11. **Hope/wish rate** ($r_{\text{hope}}$): hope-related tokens (e.g., *hope, wish, hoping*) (10).
12. **Cognitive-process rate** ($r_{\text{cog}}$): cognitive/appraisal terms (e.g., *think, know, understand, because, realize, wonder, remember*) (10, 33).

All biomarkers are computed deterministically from participant-authored text using (i) regex-based tokenisation, (ii) lexicon and phrase counting, and (iii) rate normalisation by token count. The lexicons are lightweight and editable, supporting inspection and sensitivity analyses (e.g., expanding dictionaries or replacing them with validated resources such as LIWC-style lexicons in future work).

### B Ethics approval and participant consent

This study was conducted in accordance with the ethical standards of the University of Hertfordshire. Ethical approval was granted by the University of Hertfordshire under protocol number **SPECS/SF/UH/05493**.

All participants were recruited on a voluntary basis and provided informed consent prior to participation. Due to ethical restrictions, no demographic information—such as age, gender, ethnicity, or place of birth—was collected. All data were handled in accordance with institutional data protection policies and were used exclusively for research purposes.

### C Sensitivity analysis for the SVM regularisation parameter

To assess sensitivity to the SVM regularisation parameter, we repeated the participant-wise LOOCV pipeline across multiple C values (C ∈{0.5,1,2,5,10,20}), while keeping all other components unchanged. As shown in Table C1, performance remained broadly stable across these settings, supporting the robustness of the main findings to the choice of C.

**Table C1:** Sensitivity analysis of the SVM regularisation parameter C under the same participant-wise LOOCV pipeline used in the main experiments.

C value	Accuracy	Macro-F1
0.5	0.69	0.67
1.0	0.69	0.67
2.0	0.70	0.67
5.0	0.70	0.67
10.0	0.70	0.67
20.0	0.70	0.67

## References

[1] Santomauro DF Mantilla Herrera AM Shadid J Zheng P Ashbaugh C Pigott DM, et al. Global prevalence and burden of depressive and anxiety disorders in 204 countries and territories in 2020 due to the COVID-19 pandemic. Lancet. (2021) 398:1700–12. 10.1016/S0140-6736(21)02143-7 34634250 PMC8500697

[2] World Health Organization. Data from: Anxiety disorders. Fact sheet (2025). Available online at: https://www.who.int/news-room/fact-sheets/detail/anxiety-disorders (accessed January 20, 2026).

[3] World Health Organization. Data from: Mental disorders. Fact sheet (2025). Available online at: https://www.who.int/news-room/fact-sheets/detail/mental-disorders (accessed January 20, 2026).

[4] National Institute for Health and Care Excellence (NICE). Data from: Generalised anxiety disorder and panic disorder in adults: management (CG113). Clinical guideline. Last updated 2020 (2011) (accessed January 20, 2026).

[5] First MB Williams JBW Karg RS Spitzer RL, Structured Clinical Interview for DSM-5 Disorders, Clinician Version (SCID-5-CV): Administration Booklet. Arlington, VA: American Psychiatric Association Publishing (2016).

[6] Lecrubier Y Sheehan DV Weiller E Amorim P Bonora I Harnett Sheehan K, et al. The Mini International Neuropsychiatric Interview (MINI). a short diagnostic structured interview: reliability and validity according to the CIDI. Eur Psychiatry. (1997) 12:224–31. 10.1016/S0924-9338(97)83296-8

[7] Sheehan DV Lecrubier Y Sheehan KH Amorim P Janavs J Weiller E, et al. The Mini-International Neuropsychiatric Interview (M.I.N.I.): The development and validation of a structured diagnostic psychiatric interview for DSM-IV and ICD-10. J Clin Psychiatry. (1998) 59:22–33. 9881538

[8] Malgaroli M Hull TD Schultebraucks K Palmisano J Litvinova A Huang X, et al. Natural language processing for mental health interventions: a systematic review and research framework. Transl Psychiatry. (2023) 13:309. 10.1038/s41398-023-02592-2 37798296 PMC10556019

[9] Zhang D Teo AR Wu M Zhang J Lim E Tan Y. Natural language processing applied to mental illness detection: a narrative review. npj Digit Med. (2022) 5:46. 10.1038/s41746-022-00589-7 35396451 PMC8993841

[10] Tausczik YR Pennebaker JW. The psychological meaning of words: LIWC and computerized text analysis methods. J Lang Soc Psychol. (2010) 29:24–54. 10.1177/0261927X09351676

[11] Al-Mosaiwi M Johnstone T. Linguistic markers of psychological states: the case of absolutist words. Clin Psychol Sci. (2018) 6:529–42. 10.1177/2167702617747074 30886766 PMC6376956

[12] Rook KS Rajkumar K. Linguistic markers of generalized anxiety disorder in spontaneous speech. J Anxiety Disord. (2022) 86:102509. 10.1016/j.janxdis.2022.102509

[13] Devlin J Chang M Lee K Toutanova K. BERT: pre-training of deep bidirectional transformers for language understanding. In *Proceedings of the 2019 Conference of the North American Chapter of the Association for Computational Linguistics: Human Language Technologies (NAACL-HLT)* (2019). p. 4171–86. 10.18653/v1/N19-1423

[14] Moëll B Sand Aronsson F. High-accuracy prediction of mental health scores from English BERT embeddings trained on LLM-generated synthetic self-reports: a synthetic-only method development study. Front Digit Health. (2025) 7:1694464. 10.3389/fdgth.2025.1694464 41586203 PMC12824020

[15] Mineur L Horstmann S Arslan B Andreou C Heide M Eickhoff S, et al. Neutral sentiment on patient’s speech can predict the depressive symptom severity transdiagnostically. J Affect Disord. (2025) 391:119990. 10.1016/j.jad.2025.119990 40730289

[16] Poldrack RA Huckins G Varoquaux G. Establishment of best practices for evidence for prediction: a review. JAMA Psychiatry. (2020) 77:534–40. 10.1001/jamapsychiatry.2019.3671 31774490 PMC7250718

[17] Saeb S Lonini L Jayaraman A Mohr DC Kording KP. The need to approximate the use-case in clinical machine learning. Gigascience. (2017) 6:gix019. 10.1093/gigascience/gix019 PMC5441397 28327985

[18] Bayer M Kaufhold M-A Reuter C. A survey on data augmentation for text classification. ACM Comput Surv. (2022) 55:1–39. 10.1145/3544558

[19] Wei J Zou K. EDA: easy data augmentation techniques for boosting performance on text classification tasks. *arXiv* (2019).

[20] Kang T Perotte A Tang Y Ta C Weng C. UMLS-based data augmentation for natural language processing of clinical research literature. J Am Med Inform Assoc. (2021) 28:812–23. 10.1093/jamia/ocaa309 33367705 PMC7973470

[21] Huang G Li Y Jameel S Long Y Papanastasiou G. From explainable to interpretable deep learning for natural language processing in healthcare: How far from reality? Comput Struct Biotechnol J. (2024) 24:362–73. 10.1016/j.csbj.2024.05.004 38800693 PMC11126530

[22] Marian V Kaushanskaya M. Words, feelings, and bilingualism: cross-linguistic differences in emotionality of autobiographical memories. Ment Lex. (2008) 3:72–90. 10.1075/ml.3.1.06mar 19966924 PMC2788822

[23] Yang C Li X Chen Y Zhang X Luo L Gao S. Emotion-dependent linguistic features of autobiographical memory of different specificity. Lang Cogn. (2025) 17:e83. 10.1017/langcog.2025.10039

[24] European Union. Data from: Artificial intelligence act (regulation (eu) 2024/1689): Article 12 (record-keeping). AI Act Service Desk summary of official text (2024). Available online at: https://ai-act-service-desk.ec.europa.eu/en/ai-act/article-12 (accessed January 20, 2026).

[25] U.S. Food and Drug Administration (FDA). Data from: Transparency for machine learning-enabled medical devices: guiding principles. Web page (2024). Available online at: https://www.fda.gov/medical-devices/software-medical-device-samd/transparency-machine-learning-enabled-medical-devices-guiding-principles (accessed January 20, 2026).

[26] World Health Organization. Data from: Ethics and governance of artificial intelligence for health. WHO guidance (2021). Available online at: https://www.who.int/publications/i/item/9789240029200 (accessed January 20, 2026).

[27] DeVries T Taylor GW. Dataset augmentation in feature space. *arXiv* [Preprint] *arXiv:1702.05538* (2017).

[28] Cheung T-H Yeung D-Y. Modals: modality-agnostic automated data augmentation in the latent space. In *International Conference on Learning Representations* (2020).

[29] Cortes C Vapnik V. Support-vector networks. Mach Learn. (1995) 20:273–97. 10.1023/A:1022627411411

[30] Schölkopf B Smola AJ, Learning with Kernels: Support Vector Machines, Regularization, Optimization, and Beyond. Cambridge, MA: MIT Press (2002).

[31] Cao J. Wang M. Li Y. Zhang Q. Bagci U.. Improved support vector machine classification algorithm based on adaptive feature weight updating in the Hadoop cluster environment. PLoS ONE. (2019) 14:e0215136. 10.1371/journal.pone.0215136 30970014 PMC6457544

[32] Guo C-Y Chou Y-C. A novel machine learning strategy for model selections - stepwise support vector machine (StepSVM). PLoS ONE. (2020) 15:e0238384. 10.1371/journal.pone.0238384 32853243 PMC7451646

[33] Pennebaker JW Mehl MR Niederhoffer KG. Psychological aspects of natural language use: our words, our selves. Annu Rev Psychol. (2003) 54:547–77. 10.1146/annurev.psych.54.101601.145041 12185209

[34] AssemblyAI. Data from: Speech-to-text (2025). https://www.assemblyai.com/products/speech-to-text (accessed March 22, 2026).

[35] Clark DB Donovan JE. Reliability and validity of the Hamilton anxiety rating scale in an adolescent sample. J Am Acad Child Adolesc Psychiatry. (1994) 33:354–60. 10.1097/00004583-199403000-00009 8169180

[36] Inoue T Masuda T Sano F Maruyama H. Lurasidone for bipolar I depression with comorbid anxiety symptoms: post-hoc-analysis of randomized, placebo-controlled studies. J Affect Disord. (2025) 385:119348. 10.1016/j.jad.2025.05.008. 40334859

[37] Tohen M Calabrese J Vieta E Bowden C Gonzalez-Pinto A Lin D, et al. Effect of comorbid anxiety on treatment response in bipolar depression. J Affect Disord. (2007) 104:137–46. 10.1016/j.jad.2007.03.014 17512607

[38] Hui B Yang J Cui Z Yang J Liu D Zhang L, et al. Qwen2. 5-coder technical report. *arXiv* [Preprint] *arXiv:2409.12186* (2024).

[39] Zhang Y Lyu H Liu Y Zhang X Wang Y. Natural language processing applied to mental illness detection: a systematic review. IEEE J Biomed Health Inform. (2022) 26:1022–36. 10.1109/JBHI.2021.3104490

